# Current Understanding of Host Genetics of Otitis Media

**DOI:** 10.3389/fgene.2019.01395

**Published:** 2020-02-07

**Authors:** Ruishuang Geng, Qingzhu Wang, Eileen Chen, Qing Yin Zheng

**Affiliations:** ^1^ College of Special Education, Binzhou Medical University, Yantai, China; ^2^ Department of Otolaryngology, Guangdong Second Provincial General Hospital, Guangzhou, China; ^3^ Department of Otolaryngology, Case Western Reserve University, Cleveland, OH, United States

**Keywords:** otitis media, genetics, susceptibility, immunity, inflammatory response, host, pathogenicity

## Abstract

The pathogenesis of otitis media (OM), an inflammatory disease of the middle ear (ME), involves interplay between many different factors, including the pathogenicity of infectious pathogens, host immunological status, environmental factors, and genetic predisposition, which is known to be a key determinant of OM susceptibility. Animal models and human genetics studies have identified many genes and gene variants associated with OM susceptibility: genes that encode components of multiple signaling pathways involved in host immunity and inflammatory responses of the ME mucosa; genes involved in cellular function, such as mucociliary transport, mucin production, and mucous cell metaplasia; and genes that are essential for Eustachian tube (ET) development, ME cavitation, and homeostasis. Since our last review, several new mouse models with mutations in genes such as *CCL3*, *IL-17A,* and *Nisch* have been reported. Moreover, genetic variants and polymorphisms in several genes, including *FNDC1, FUT2, A2ML1, TGIF1*, *CD44,* and *IL1-RA* variable number tandem repeat (VNTR) allele 2, have been identified as being significantly associated with OM. In this review, we focus on the current understanding of the role of host genetics in OM, including recent discoveries and future research prospects. Further studies on the genes identified thus far and the discovery of new genes using advanced technologies such as gene editing, next generation sequencing, and genome-wide association studies, will advance our understanding of the molecular mechanism underlying the pathogenesis of OM and provide new avenues for early screening and developing effective preventative and therapeutic strategies to treat OM.

## Introduction

Otitis media (OM) is an inflammatory disease of the middle ear (ME) that is most commonly caused by bacterial pathogens, such as *Streptococcus pneumoniae* (Spn), nontypeable *Haemophilus influenzae* (NTHi), and *Moraxella catarrhalis*, and is one of the most common diseases in young children. There are several types of OM, such as acute OM (AOM), chronic OM with effusion (COME), and chronic suppurative OM (CSOM). More than 80% of children under the age of three suffer at least one episode of AOM; however, only a small subset of children experience recurrent or chronic OM (COM), and the reason for this remains unclear (Kong and Coates, 2009). Although most cases of OM are resolved by the age of six, prolonged ME inflammation can lead to hearing loss and other complications in some cases.

The pathogenesis of OM is known to be multifactorial, involving pathogen virulence, host immune status, genetic predisposition, and environmental factors, alongside other risk factors that affect the occurrence of COM, such as allergy/atopy, prior upper respiratory tract viral infection, early or recurrent AOM, and passive smoking (Zhang et al., 2014). Genetics has also been shown to play a critical role in host susceptibility to OM; therefore, identifying genetic loci that are associated with OM could help to elucidate potential disease mechanisms and develop effective therapies. Many host genes identified and associated with OM have been reviewed before (Rye et al., 2011a; Kurabi et al., 2016; Bhutta et al., 2017b; Lin et al., 2017). In this review, we provide an update on the identification of new host genes, make progress in our understanding of previously identified genes in OM predisposition, and discuss the prospects for future research in this field.

## Innate Immune and Inflammatory Responses in OM

The epithelial lining of the ME possesses several defense mechanisms; for instance, ME epithelial cells secret mucin and other defense molecules (e.g. defensins, interferons, lactoferrin, and nitric oxide) to attack and trap pathogens, particles, and dead cells, which are conveyed toward the nasopharynx *via* the Eustachian tube (ET) and cleared from the ME by the constant unidirectional beating of the cilia of ciliated epithelial cells. ME epithelial cells also express pattern recognition receptors (PRRs), such as Toll-like receptors (TLRs) and Nod-like receptors (NLRs), which recognize bacterial pathogens by binding to pathogen-associated molecular patterns (PAMPs) on their surface. The binding of PRRs and PAMPs activates downstream MAPK or NFκB signaling cascades to induce the expression and activation of pro-inflammatory transcription factors, such as NFκB and interferon-regulatory factors (IRFs). These transcription factors translocate to nucleus and induce the production and release of inflammatory cytokines and chemokines, which recruit and activate neutrophils, macrophages, and monocytes that destroy and clear invading bacterial pathogens (Leichtle et al., 2011; Kurabi et al., 2016).

### Genes Involved in Inflammatory Responses in OM

During the past decade, considerable progress has been made in our understanding of the fundamental molecular mechanisms underlying the role of innate immunity and inflammatory responses in OM (Kurabi et al., 2016). The innate immune system plays important roles in the initiation of inflammation, clearance of invading pathogens, and recovery in AOM. Many genes have been identified that are involved in immunity and inflammatory responses in OM, and their functions have been studied in animal models. The most important discoveries of mouse models and gene association studies are briefly summarized and discussed below.

Pro-inflammatory molecules, such as TNF-α, IL-1β, and C-C motif chemokine ligand 3 (CCL3), play key roles in the recruitment of inflammatory cells into the ME and the activation of these cells for microbial clearance. Mice deficient in pro-inflammatory molecules, such as TNF-α and CCL3, displayed diminished but prolonged leukocyte recruitment, defective macrophage function, and failure to clear NTHi from the ME cavity (Leichtle et al., 2010; Deniffel et al., 2017). Moreover, exogenous CCL3 can restore phagocytosis and fully restore OM recovery, suggesting that CCL3 acts downstream of TNF-α (Leichtle et al., 2010). These data pinpoint the essential roles of pro-inflammatory molecules in the initiation and recovery in AOM.

Mouse mutants deficient in PRRs (TLR2, TLR4, and TLR9), NLRs [apoptosis-associated speck-like protein containing a caspase-activating and recruitment domain (ASC)], and adaptor proteins (MyD88 and TRIF) display reduced production and maturation of pro-inflammatory cytokines, such as IL-1 and TNF-α, which leads to reduced leukocyte recruitment to the ME and, more profoundly, persistent inflammation with impaired bacterial clearance, and this is consistent with their roles in mediating the production of pro-inflammatory cytokines in response to pathogens and in the recovery of AOM (Hirano et al., 2007; Hernandez et al., 2008; Han et al., 2009; Leichtle et al., 2009a; Leichtle et al., 2009b; Leichtle et al., 2012). Similarly, in children, genetic polymorphisms in TLR2, TLR4, and the TLR co-receptor CD14 have been found to be associated with an increased incidence of OM, while TLR4 also plays a role in acquired adaptive mucosal immunity in the ME (Wiertsema et al., 2006; Emonts et al., 2007; Hafren et al., 2015; Toivonen et al., 2017).

The active form of IL-1β is a cleavage product formed by the inflammasome, a multi-protein complex that consists of the NLRs ASC and pro-caspase 1. ASC-deficient mutants display a lack of IL-1β maturation in the ME, reduced leukocyte recruitment and infiltration in the ME cavity, and reduced NTHi phagocytosis (Kurabi et al., 2015). Moreover, ASC deficiency increases the degree and duration of mucosal epithelial hyperplasia in the ME and delays bacterial clearance in the infected ME cavity (Kurabi et al., 2015). In a microphage cell model infected with Spn, IL-1β and TNF-α secretion were significantly reduced by treatment with inhibitors of c-Jun N-terminal kinase (JNK) or spleen tyrosine kinase (Syk). Furthermore, it has been demonstrated that JNK is required for ASC oligomerization and caspase-1 activation, and that JNK activation *via* phosphorylation is regulated by Syk (Feng et al., 2018) with similar results having also been obtained in neutrophils upon Spn infection. In addition to JNK, neutrophil serine proteases have also been found to participate in IL-1β secretion by regulating ASC oligomerization and caspase-1 activation (Zhang et al., 2019).

It has also been shown that IL-17A levels are significantly upregulated in ME fluid during AOM. Wang et al. reported that IL-17A promotes neutrophil recruitment to the ME cavity and neutrophil apoptosis for bacterial clearance *via* the p38 MAPK signaling pathway during Spn infection (Wang et al., 2014a). The same group found that IL-17A also induces ME injury since *IL-17A* knockout (KO) mice display less severe pathological changes in their ME and lower pro-inflammatory cytokine and myeloperoxidase (MPO) levels. Furthermore, the group showed that neutrophil MPO production is mediated by the p38 MAPK signaling pathway (Wang et al., 2017).

In summary, the innate immune system mediated by TLR, NLR, and their downstream effectors and signaling pathways play critical roles in the production of pro-inflammatory molecules in response to pathogens for the recruitment of leukocytes into ME and recovery of AOM. Other signaling pathways, such as JNK and MAPK, also participate in the initiation of inflammation and ME injury, which may be caused by prolonged existence of leukocytes and elevated level of myeloperoxidase in the ME.

### Genes Involved in Anti-Inflammatory Responses in OM

While pro-inflammatory responses fight infection and damage host tissue, they are balanced by anti-inflammatory responses that are thought to protect against host tissue damage and initiate repair and healing to restore tissue homeostasis. Both pro- and anti-inflammatory cytokines and cytokine signaling genes are rapidly upregulated in response to NTHi, as shown by transcriptome assays performed during a complete episode of AOM (Hernandez et al., 2015). IL-6, which acts as both a pro- and anti-inflammatory cytokine, is significantly upregulated 3–6 h after NTHi inoculation, with its decline followed by significant increases in other pro-inflammatory cytokines (IL-1 β and TNF-α) and anti-inflammatory cytokines (IL-1 receptor antagonist (IL-1RA) and IL-10) (Hernandez et al., 2015). It has been found that IL-10 is associated with OM and plays a critical role in protecting the cochlea from inflammation-mediated tissue damage by negatively regulating *MCP-1/CCL2* expression (Ilia et al., 2014; Woo et al., 2015). Deubiquitinase cylindromatosis (CYLD) has been found to suppress NTHi-induced inflammation by inhibiting the expression of the key pro-inflammatory chemokine IL-8 *via* the MAP kinase phosphatase 1 (MKP-1)–dependent inhibition of extracellular signal-regulated kinase (ERK) (Wang et al., 2014b). In a recent study, Zivkovic et al. found that the *IL-1RA* VNTR allele 2 was associated with a chronic OM patient in Serbia and suggested that the allele is associated with higher IL-1RA levels, consistent with the increase in IL-1RA observed in mouse models of OM (Zivkovic et al., 2018).

*Several TGF*β signaling pathway genes have been associated with AOM in humans, and the TGFβ signaling pathway has been shown to be involved in anti-inflammatory function in mouse models of OM (Ilia et al., 2014; Rye et al., 2014). The immunomodulatory gene transforming growth interacting factor 1 (TGIF1) is a negative regulator of the TGFβ signaling pathway, and *Tgif1* knockout mice develop spontaneous COME, which is characterized by significant thickening of the ME mucosa lining and goblet cell population expansion (Tateossian et al., 2013). In addition, studies in *Fbxo11-* and *Evi1-deficient* mouse models have indicated that OM is *associated with defects in the regulation of* TGFβ signaling. Evi1 is known to repress TGFβ signaling in several pathological processes by interacting with different proteins and negatively regulate NTHi-induced inflammation by inhibiting NFκB activity (Kurokawa et al., 1998; Izutsu et al., 2001; Sato et al., 2008; Xu et al., 2012). Moreover, Evi1 dominant mutations lead to the spontaneous development of OM in mice under specific pathogen-free conditions (Parkinson et al., 2006; Hood et al., 2016). *Fbxo11 is also involved in* TGFβ signaling by regulating phosphoSmad2 levels in the epithelial cells of palatal shelves, while a recent report showed that *Fbxo11^Jf^^/+^* mutations cause failed mesenchymal regression during bulla cavitation, which may be the underlying cause of OM (Tateossian et al., 2009; Del-Pozo et al., 2019a). FBXO11 and TGIF1 have also been associated with COM in humans (Segade et al., 2006; Rye et al., 2011b; Bhutta et al., 2017a), and the involvement of EVI1, FBXO11, and TGIF1 in the development of COM may be mediated *via* vascular endothelial growth factor (VEGF) signaling, which was found to be upregulated in the leukocytes of these mutant mice during the bulla fluid response to inflammatory hypoxia (Cheeseman et al., 2011). Furthermore, VEGF signaling has also been demonstrated in the effusions of children with COME, while NFκB has been detected in the mucosa of patients with CSOM (Sekiyama et al., 2011; Jesic et al., 2014). These data further suggest the regulation of ME inflammation involves complex interactions between several signaling pathways. Continuing study is needed to reveal the molecular mechanism underlying the pathogenesis of COM.

### Other Genes Involve in Innate Immune and Inflammatory Response in OM

BPIFA1 (SPLUNC1), which is abundant in the mammalian nasal, oral, and respiratory mucosa, has broad-spectrum antimicrobial activity and can act as a chemoattractant that recruits macrophages and neutrophils to the site of infection (Sayeed et al., 2013). In mice, BPIFA1 is highly expressed in the surface epithelium, submucosal glands in the ME, and ET, while BPIFA1-deficient mice display an increased COM frequency, as characterized by the accumulation of neutrophils, proteinaceous fluid, and mucus in the ME and extensive remodeling of the ME walls (Bartlett et al., 2015). Recently, Mulay et al. found that *Bpifa1* deletion in Evi1^Jbo/+^ mice significantly worsened the OM phenotype, thickening the ME mucosa and increased collagen deposition, without significantly increasing pro-inflammatory gene expression. The authors concluded that BPIFA1 is involved in maintaining homeostasis within the ME and its loss causes more severe OM *via* a mechanism other than the inflammatory response (Mulay et al., 2018). In this study, the deletion of *BPIFA1* alone does not increase the susceptibility to OM, which is different from the ENU *Bpifa1^−/−^* mutant. This discrepancy in the predisposition to OM may be due to the fact that the mutations are in different genetic backgrounds (Bartlett et al., 2015; Mulay et al., 2018).

Recently, Wang et al. found a multifunctional growth factor, Progranulin (PGRN), was involved in AOM in an unusual way in the study of PGRN-deficient (PGRN^-/-^) mouse model (Wang et al., 2018). After Spn inoculation, *PGRN^-/^* ^-^ mice exhibited increased macrophage recruitment in ME but delayed bacteria clearance. They found the production of CCL2 is increased, which could contribute to enhanced macrophage recruitment, whereas the delayed bacteria clearance is most likely due to impaired endocytosis capacity of macrophages (Wang et al., 2018).

Many studies have induced AOM in rodent models using Spn, NTHi, and influenzae A virus to conduct transcriptomic analyses and thereby identify genes and associated pathways involved in OM. These studies have found that expression of the inflammatory cytokines *Cxcl1, Cxcl2*, and *IL-6*, and genes involved in NFκB signaling, innate immune, and inflammatory responses are upregulated in OM. These data further support the roles of inflammatory cytokines and innate immune response in AOM (MacArthur et al., 2013; Hernandez et al., 2015). Genes that are important for craniofacial structure and ME cellular function in OM.

The coordinated movement of cilia toward the pharynx *via* the ET is essential for trapping pathogens and mucociliary clearance. Many OM mouse models are characterized by gene mutations that cause defects in craniofacial anatomy or ME cellular function, including mucin production, ciliated cell function, ET structure and function, ME cavitation, and mucosal hyperplasia. Many of these mouse mutants spontaneously develop COME and thus can serve as good models for studying COME (Bhutta et al., 2017b; Lin et al., 2017).

In the past few years, continuing efforts have been made to identify new genes and further characterize existing mouse models. Mutations in TNF-like ligand ectodysplasin (Eda) and its receptor, Edar, result in the impaired development or loss of submucosal glands, leading to reduced ET gating and the ascension of bacteria and foreign body particles into the ME cavity as well as reduced mucociliary clearance in mice and rats (Azar et al., 2016; Del-Pozo et al., 2019b). Mutations in the EYA transcriptional coactivator and phosphatase 4 *(Eya4) and Fbxo11, which cause delayed or failed* mesenchyme regression during ME cavitation, have also been associated with OM (Depreux et al., 2008; Del-Pozo et al., 2019a). T-box transcription factor 1 (Tbx1) deficiency disrupts the function of the muscles that control ET function (Fuchs et al., 2015; Funato and Yanagisawa, 2018), while OM model mice carrying mutations in the cell adhesion protein Cdh11 display ME cavitation defects (Kiyama et al., 2018). Further study of many of previous identified genes in mouse models is needed to elucidate the cellular and molecular mechanism of these genes in OM.

## Recently Identified Genes Associated With OM

In the past decade, a number of genetic loci, such as those at 10q26.3, 19q13.43, 17q12, 10q22.3, and 2q31.1, and genes including *A2ML1, BPIFA1, CAPN14, GALNT14*, *FBXO11*, *FNDC1, FUT2*, and *TGIF1,* have been reported to be associated with OM (Daly et al., 2004; Casselbrant et al., 2009; Chen et al., 2011; Rye et al., 2011b; Rye et al., 2012; Allen et al., 2013; Rye et al., 2014; Santos-Cortez et al., 2015; Einarsdottir et al., 2016; Santos-Cortez et al., 2016; van Ingen et al., 2016; Bhutta et al., 2017a; Santos-Cortez et al., 2018). In addition, several genes have been identified as associated with childhood ear infection *via* a genome-wide association study (GWAS), such as *FUT2, TBX1, ABO, MKX*, *FGF3*, *AUTS2, CDHR3, and PLG (* Tian et al., 2017 *).* Among these, *FUT2 and TBX1 were associated with OM by separate studies;* however, many of these associations did not reach the genome-wide significance threshold (*p* < 5 × 10^−8^), therefore their *involvement in OM requires further validation. Fortunately,* during the past three years, an increasing number of genes have been identified by GWAS, exome sequencing, linkage analysis, and the use of mouse mutants.

The alpha-2-macroglobulin-like 1 (*A2ML1*) gene encodes an ME-specific protease inhibitor with 41% identity and 59% similarity with alpha-2-macroglobulin (*A2M*), an inflammatory marker of the ME and oral cavity. A number of rare *A2ML1* variants have been associated with OM susceptibility in indigenous Filipino and in European- and Hispanic-American children (Santos-Cortez et al., 2015; Santos-Cortez et al., 2016; Larson et al., 2019). An RNAseq analysis has shown that *A2ML1* upregulation *is correlated with the* differential expression of genes in the keratinocyte and epidermal cell differentiation pathways, further suggesting that these rare *A2ML1* variants play a role in ME mucosal pathology.

*FUT2*, which encodes alpha-(1,2)-fucosyltransferase, is a human secretion gene that controls the expression of the Lewis and ABO(H) antigens on the mucosal epithelia, *via* which bacterial pathogens bind (Goto et al., 2016). GWAS identified *FUT2 as a potential susceptibility gene for ear infections in children (* Tian et al., 2017 *), while* Santos-Cortez *et al. found that common and rare FUT2* variants confer susceptibility to recurrent/chronic OM in patients from various ethnicities. FUT2 likely modulates the ME microbiome by regulating A antigen levels in epithelial cells (Santos-Cortez et al., 2018).

Van Ingen et al. identified that the fibronectin type III domain containing 1 (*FNDC1*) gene is significantly associated with AOM *via* GWAS (van Ingen et al., 2016). Previous reports have suggested that FNDC1 is involved in multiple cellular processes, including inflammation. The AOM-associated *FNDC1* variants were correlated with the methylation status of the *FNDC1* gene and their association surpassed the threshold of genome-wide significance and was replicated in an independent cohort. Moreover, Fndc1 is expressed in the ME tissue of mice and its expression upregulated upon lipopolysaccharide treatment, which is known to potently induce inflammation and stimulate TGF-β, TNF-α, and IL-1 signaling. Thus, these studies imply that *FNDC1* may be involved in the pathogenesis of OM by modulating immunity or inflammatory responses (van Ingen et al., 2016).

CD44 is a transmembrane glycoprotein receptor for hyaluronic acid that is widely expressed on the surface of leukocytes, endothelial cells, epithelial cells, fibroblasts, and keratinocytes and is involved in cell-cell interactions, cell adhesion, and migration. Mice deficient in CD44 exhibit reduced early mucosal hyperplasia and leukocyte recruitment and delayed bacterial clearance, suggesting it plays a critical role in the cellular function of leukocytes and epithelial cells as wells as in the pathogenesis and recovery of OM (Lim et al., 2019).

Nischarin is a cytosolic protein that is involved in the regulation of cell motility, cell invasion, vesicle maturation, and tumor suppression by interacting with multiple interacting partners. Crompton et al. reported that mice with a mis-sense mutation in the *Nisch* gene spontaneously develop OM, with progression to chronic OM evidenced by histological examinations (Crompton et al., 2017). Moreover, the mutant mice exhibit serous or granulocytic effusions, become increasingly macrophage- and neutrophil-rich with age, and develop a thickened, inflamed mucoperiosteum. Significant genetic interactions have also been observed between *Nisch* and *Itga5* mutations in the penetrance and severity of chronic OM, while immunohistochemical staining and protein expression analysis have implicated PAK1, RAC1, and downstream LIM domain kinase 1 (LIMK1) and NFκB pathway signaling in the development of chronic OM (Crompton et al., 2017). Further study on the molecular pathways involve these recently identified genes could provide insight into the pathogenesis of OM.

## MicroRNAs and OM

Several reports have suggested that miRNAs are involved in the pathogenesis of OM. Song et al. identified 15 differentially expressed miRNAs from human ME epithelial cells (HMEECs) treated with lipopolysaccharides (LPS), which are a cell wall component of gram-negative bacteria. The predicted target genes of these miRNAs are involved in developmental processes, regulating cell growth, innate immune responses, acute inflammatory responses, the IκB kinase/NFκB cascade, complement activation, cell communication, and cell differentiation, among others (Song et al., 2011). Val et al. detected miRNAs from chronic OM ME effusions and identified five miRNAs (miR-378a-3p + miR-378i, miR-200a-3p, miR-378g, miR30d-5p, and miR-222-3p) that were significantly induced in exosomes from HMEECs exposed to NTHi lysates, all of which are known to target innate immunity genes (Val et al., 2018). Associations between miRNAs and OM have also been reported in humans; for instance, miR-146 expression is increased in the ME of OM patients and *in vitro* cultured ME epithelial cells stimulated with proinflammatory cytokines. Therefore, identifying miRNA target genes and their downstream pathways could provide new insights into OM (Samuels et al., 2016).

## Concluding Remarks

During the past decade, mutagenesis and mutant characterization studies in mouse models have identified many genes as predisposing factors to OM which are predominantly involved in host immune and inflammatory responses, cellular function in mucin production, mucociliary transport, and the development of the ME cavity and craniofacial structure. Genetic studies in human patients have identified far fewer genes and loci that are significantly associated with OM due to small patient sample sizes, poor phenotyping, and more complex genetic polymorphisms. However, there has been some consistency between the genes identified by human and mouse genetic studies, with polymorphisms or variants of the human orthologs of mouse genes, such as *TLR2, TLR4, FBXO11, and BPIFA1*, also found to be significantly associated with OM in humans. Moreover, some OM-associated syndromic disease genes have also been identified in mice.

Studies in both human and mouse models have shown that the host innate immune system plays crucial roles in the pathogenesis and recovery of AOM. Deficiency in PRRs and downstream signaling molecules that affect pro-inflammatory factor production delay OM recovery, while mouse mutants with ME cellular function defects and craniofacial abnormalities often spontaneously develop chronic OM. Chronic OM has also been associated with genetic polymorphisms or mutations in genes involved in or mediated by innate immunity, VEGF, *and TGF*β signaling pathways, such as *Tlr4, Evi1, Nisch, Bpifa1, Tgif1*, and *Fbxo11*.

Mouse mutants have been shown to recapitulate many features of human chronic OM, including ME leukocyte infiltration, mucosal hyperplasia, and the production of mucus-rich effusions, thus serve as excellent models for studying the pathology and mechanism underlying the pathogenesis and recovery of OM. The human counterparts of these mouse genes associated with OM predisposition should be investigated as candidate genes for genetic linkage or association studies in human OM patients of different ancestries. However, the majority of studies in animal models are phenotypic or pathophysiological, and little is known about the disease-related pathways or molecular mechanisms underlying the pathogenesis of chronic OM. Thus, further studies on these animal models are necessary.

There are several limitations to the study of OM using mouse models. Firstly, they may not fully recapitulate the features of human OM; for example, there is no mouse model of CSOM. Secondly, the human counterparts of COME causal genes in mice, such as those causing syndromic disease and craniofacial abnormalities, may not account for the high prevalence of OM in humans as the correlation between ET abnormalities and chronic OM susceptibility in young children remains unclear (Sade et al., 1986; Takasaki et al., 2007). Instead, craniofacial abnormalities may be more associated with chronic OM in adult patients (Dinc et al., 2015; Nemade et al., 2018). OM susceptibility is likely to be polygenic. Thirdly, many of these mouse models have been obtained by mutagenesis in isogenic inbred lines housed in clean facilities, whereas the human population has huge genetic variation and polymorphisms, experiences diverse living conditions, and is exposed to different environments. Studying OM gene function in outbred laboratory animals may be a better approach. Nevertheless, mice still serve as good models for studying the etiology, pathophysiology, and recovery process of human OM. Therefore, the genes that have been associated with OM in humans but failed to exceed genome-wide significance thresholds or could not be replicated in different cohorts should be further investigated in mice using reverse genetics tools, such as gene editing.

In the past three years, promising results have been obtained by using GWAS, exome sequencing, and linkage analysis to identify human genes associated with OM. Genetic variants and polymorphisms in several genes, such as *FNDC1, FUT2, A2ML1, TGIF1,* and *CD44*, have been identified as significantly associated with OM, confirming that the genome-wide significance of genetic associations can be improved by increasing the size of the study group. With continuing advancements in genetic analysis technologies and experimental design, such as increased sample size, more defined phenotyping, and less diversity in the ancestry of the study group, future studies should be able to identify more novel OM-predisposing genes to advance our understanding of the mechanism underlying the pathogenesis and recovery of OM. This information would, in turn, provide new options for efficient diagnosis and developing effective therapies that target the specific etiology of OM.

## Author Contributions

RG, QW, EC, and QZ collected data and wrote the manuscript. All authors have read and approved the submitted form.

## Funding

This work was supported by the National Institute of Health (R01DC015111 and R21DC005846); the National Natural Science Foundation of China (81530030, 35281873679, 81500797, and 81700902); the Taishan Scholar Foundation (ZR2019PH06); and Binzhou Medical University start-up fund.

## Conflict of Interest

The authors declare that the research was conducted in the absence of any commercial or financial relationships that could be construed as a potential conflict of interest.
